# A Naturalistic Study of Youth Referred to a Tertiary Care Facility for Acute Hypomanic or Manic Episode

**DOI:** 10.3390/brainsci10100689

**Published:** 2020-09-29

**Authors:** Gabriele Masi, Stefano Berloffa, Pietro Muratori, Maria Mucci, Valentina Viglione, Arianna Villafranca, Emanuela Inguaggiato, Valentina Levantini, Francesca Placini, Chiara Pfanner, Giulia D’Acunto, Francesca Lenzi, Francesca Liboni, Annarita Milone

**Affiliations:** IRCCS Stella Maris, Scientific Institute of Child Neurology and Psychiatry, 56028 Calambrone-Pisa, Italy; sberloffa@fsm.unipi.it (S.B.); pmuratori@fsm.unipi.it (P.M.); mmucci@fsm.unipi.it (M.M.); vviglione@fsm.unipi.it (V.V.); avillafranca@fsm.unipi.it (A.V.); einguaggiato@fsm.unipi.it (E.I.); valentina.levantini@unifi.it (V.L.); fplacini@fsm.unipi.it (F.P.); cpfanner@fsm.unipi.it (C.P.); gdacunto@fsm.unipi.it (G.D.); flenzi@fsm.unipi.it (F.L.); fliboni@fsm.unipi.it (F.L.); annarita.milone@fsm.unipi.it (A.M.)

**Keywords:** bipolar disorders, children, adolescents: ADHD, substance use disorder, suicidality, anxiety disorders, externalizing disorders

## Abstract

Background: Bipolar Disorders (BD) in youth are a heterogeneous condition with different phenomenology, patterns of comorbidity and outcomes. Our aim was to explore the effects of gender; age at onset (prepubertal- vs. adolescent-onset) of BD; and elements associated with attention deficit hyperactivity disorder (ADHD) and Substance Use Disorder (SUD) comorbidities, severe suicidal ideation or attempts, and poorer response to pharmacological treatments. Method: 117 youth (69 males and 57 females, age range 7 to 18 years, mean age 14.5 ± 2.6 years) consecutively referred for (hypo)manic episodes according to the *Diagnostic and Statistical Manual of Mental Disorders*, 54th ed (DSM 5) were included. Results: Gender differences were not evident for any of the selected features. Prepubertal-onset BD was associated with higher rates of ADHD and externalizing disorders. SUD was higher in adolescent-onset BD and was associated with externalizing comorbidities and lower response to treatments. None of the selected measures differentiated patients with or without suicidality. At a 6-month follow up, 51.3% of the patients were responders to treatments, without difference between those receiving and not receiving a psychotherapy. Clinical severity at baseline and comorbidity with Conduct Disorder (CD) and SUD were associated with poorer response. Logistic regression indicated that baseline severity and number of externalizing disorders were associated with a poorer outcome. Conclusions: Disentangling broader clinical conditions in more specific phenotypes can help timely and focused preventative and therapeutic interventions.

## 1. Introduction

Even if bipolar disorder (BD) is a well-established clinical picture in adults, its presentation in children and early adolescents is frequently “atypical”, compared to adult-onset presentation [1,2,3]. Formal systematic studies have led to a definition of clinical subtypes and early signs, but clinical phenotypes and boundaries of BD in youths are still debated, given the possible developmentally different presentations of the early-onset form [4,5] as well as the high rate of comorbidities [2,3]. A first clinical differentiation in manic children was between a “narrow” and a “broad” phenotype, according to the degree of fit to the full *Diagnostic and Statistical Manual of Mental Disorders*, 54th ed (DSM 5) diagnostic criteria for adult mania [4,6], but only the narrow phenotype (elated mood, euphoria and grandiosity, as well as other typical manic symptoms) has been included in the DSM 5 while the broad phenotype has been prevalently included in the new DSM 5 category of “Disruptive Mood Dysregulation Disorder” [4,5,7].

Valuable clinical information can be derived from rigorous, controlled studies, with an experimental design, controlled variables, strict exclusion criteria (i.e, severe comorbidities, substance abuse and suicidal behavior), specific and focused outcome measures, and selected treatments, however limiting the generalizability of findings to broader clinical populations. Alternatively, observational studies in realistic, nonexperimental conditions, including larger samples of unselected, consecutively referred patients with all comorbidities assessed with global measures of outcome and treated as usual with adjunctive treatments, preclude solid conclusions and may be less innovative in terms of aims and findings but are more informative in terms of generalization of results to everyday clinical practice. An integration and a comparison of data from both sources can be more clinically informative. Consistent with the design and the aims of our study, we have included in this presentation only data from naturalistic settings, which may be descriptive in term of everyday clinical practice. However, evidence from these studies is partly inconsistent, suggesting that there is room for further studies.

Available information on possible developmental differences between prepubertal- or childhood-onset BD and adolescent-onset BD, in terms of presentation, comorbidities and treatment response, is still inconsistent. Some authors [2] did not find significant differences between bipolar children and adolescents, according to gender distribution, manic symptomatology and comorbidity. Perlis et al. [8] found that bipolar patients with prepubertal-onset are at risk of a particularly severe course, greater comorbidity, recurrence and chronicity and that patients with BD onset between 13 and 18 years of age are intermediate between the prepubertal-onset and the adult-onset BD. In Biederman et al.’s study [9], childhood-onset was characterized by greater comorbidity with attention deficit hyperactivity disorder (ADHD) and by prevalent chronic course, irritable mood, and comorbidity with disruptive behavior disorders and anxiety disorders, although these features were also largely represented in adolescent patients. Masi et al. [10] found that patients with childhood-onset were more frequently males, had a chronic course, and had more frequent comorbidity with ADHD and oppositional-defiant disorder (ODD). Severity, 6-month treatment outcome, prevalent mood (elated versus irritable) and comorbid anxiety did not differentiate the two groups.

Regarding comorbidity, two broad patterns have been described, the first with ADHD and disruptive behavior disorders, such as ODD and Conduct Disorder (CD), and the second with anxiety disorders, which may define specific subtypes and developmental pathways of BD [11]. The first pattern includes preexisting ADHD [12,13,14], with rates ranging from 30% to 90%, often associated with ODD and/or conduct disorder (CD) [15,16]. The second pattern of comorbidity includes anxiety disorders, often multiple [17,18], and obsessive compulsive disorder (OCD) [19]. Multiple anxiety disorders (MAD) have been more closely related to bipolarity [16,17,20,21]. The rates of ADHD comorbidity are particularly high in prepubertal children [12,22], and are confirmed when children are assessed after removing overlapping symptoms [23]. Bipolar patients with ADHD, compared to patients without ADHD, were predominantly males and younger and had an earlier onset of BD, presenting more frequently a chronic rather than an episodic course of BD, an irritable rather than elated mood and greater psychosocial impairment [22,24].

Adolescent BD markedly increases suicidal risk [25]. The comparison of BD adolescents with or without suicidality may help to highlight possible risk factors [26], including comorbidities with ADHD [27,28] and anxiety disorders [29,30]. Further research is needed to support these findings regarding possible vulnerability factors and putative targets of timely and preventative interventions.

Substance Use Disorder (SUD) is a common comorbidity arising during the early course of BD, even before the first activated episode [31,32], and it may have a devastatingly negative effect on the clinical course and prognosis. Swendsen et al. [33], based on the findings from the U.S. National Comorbidity Survey, highlighted that mood disorders are risk factors for the subsequent onset of SUD, suggesting that early effective treatment of the primary illness is an important step in preventing the transition from use to abuse or dependence. Comorbid SUD has been associated with an earlier age of onset of BD, shortening of cycle length, delayed time to recovery, higher number of recurrences, more mixed and rapid cycling presentations, chronicity, disability, cognitive impairment and elevated mortality associated with medical decline as well as suicide; for review, see [34]. Further research in youth is needed to understand causative factors and to develop effective early intervention and prevention strategies [35].

Follow-up studies firstly supported the notion that BD in children and adolescents is associated with a more severe course and outcome [36,37]. However, predictors of treatment response in early-onset BD are not well defined. Some features, which in adult patients with BD are predictors of poor treatment response, such as baseline severity, mixed states, psychotic symptoms and comorbid SUD, are particularly frequent in youth. In the Werry and McClellan study [38], no clinical predictors of poor outcome were found, whereas the best predictors of future functioning were premorbid functioning, IQ **<** 80 and bipolar family history, suggesting a lower impact of the illness compared to factors external to the clinical picture. Some early studies [36] observed that comorbid psychopathology, mainly behavioral disorders, and, to a lesser degree, earlier age at onset and baseline clinical severity predicted a poorer outcome and, more specifically, that comorbid ADHD predicts lithium efficacy [39,40]. Other studies found that comorbidities, including ADHD, did not affect response to lithium treatment while the presence of psychotic symptoms was associated with poor lithium response [41]. In more recent studies, comorbidity with conduct disorder, anxiety disorder, psychotic symotoms ADHD, baseline clinical severity and higher number of lifetime systems-of-care for the child have been reported as possible negative predictors [42,43,44,45]. As regards psychotherapeutic interventions for youth with BD, studies found that family psychoeducational and cognitive-behavioral therapy are partially efficacious [46]. Greater results are reached when interventions that involve families, psychoeducation and skill-building are implemented in combination with pharmacotherapy [47].

The aim of the present naturalistic study, conducted in a sample of bipolar children and adolescents consecutively referred for (hypo)manic episodes in a 2-year period, was a systematic exploration on whether gender, age at onset, and ADHD and substance abuse comorbidity may influence phenomenology and outcome and to define possible elements associated to suicidality. This study also aimed to individuate possible clinical features associated to poorer response to pharmacological treatments.

## 2. Materials and Methods

### 2.1. Sample

This was a naturalistic study based on a clinical database of youth with BD consecutively referred during a 2-year period (2016–2018) for manic or hypomanic symptoms to our third-level Department of Child and Adolescent Psychiatry and Psychopharmacology, with nation-wide catchment, followed for at least 6 months and not included in previous studies. The inclusion criterion for participation was fulfillment of the DSM 5 criteria for BD, including the number of symptoms, duration and impairment, according to a Clinical Global Impression Scale (CGI-S) [48] score 4 or more (clinical severity), and Child Global Assessment Scale (C-GAS) [49] score 60 or less (functional impairment). All patients with intellectual disability were excluded. The sample consisted of 117 patients, 69 males (59.0%) and 48 females (41%), aged between 7 and 18 years, with a mean age at the time of admission of 14.5 ± 2.6 years. Thirty-nine patients (33.3%) presented a prepubertal onset of BD, before 12 years of age, while 78 (66.6%) had an adolescence onset, after 12 years.

All subjects were evaluated for current and lifetime Axis I psychiatric disorders at intake using historical information, a diagnostic interview, the Kiddie Schedule for Affective Disorders and Schizophrenia for School-Aged Children-Present and Lifetime Version (K-SADS-PL) [50], administered individually to the patients and to their parents by trained child psychiatrists. Furthermore, behavioral and social-emotional skills were assessed during interactions with peers, parents and/or examiners by trained child psychiatrists throughout the diagnostic phase. The trained child psychiatrist was the same one who participated in the subsequent assessment procedures on the same subjects, along with other examiners, and thus, he was not blind to the nature of the diagnosis. To improve the reliability and validity of the structured interview, after each interview, clinical data from each patient–parent pair were reviewed, and when subject or parent interviews endorsed bipolar diagnosis and the other did not or when other questions arose, another consultation with both patient and parent was added for further clarification in order to obtain a definitive diagnosis. In 27 out of 117 cases (23.1%), subjects and parents differed in endorsing BD and needed further consultation. Good reliability using K-SADS-PL was found, with kappa coefficients of agreement higher than 0.75 (mean k = 0.85 for all diagnoses, k value for BD = 0.82).

All patients received a naturalistic pharmacological treatment, with the following rules regarding prescriptions. Bipolar patients were primarily treated in monotherapy with a mood stabilizer, lithium or valproic acid (VPA), then with an association of both lithium and VPA, while second-generation antipsychotics (SGAs) (risperidone, olanzapine, aripiprazole and quetiapine) were used in nonresponders or as a first option only when psychotic symptoms and/or severe aggression and hostility were associated. Other medications, particularly Methylphenidate (MPH) and antidepressant Selective Serotonin Reuptake Inhibitors (SSRIs) were used when needed.

After the 6-month follow-up, patients were reassessed with the CGI-S and C-GAS and with the CGI-Improvement score (CGI-I) and were considered responders when CGI-I was 1 or 2 (very much or much improved) and C-GAS improved at least 30%; the CGI-S score was below 3, and the C-GAS was higher than 60.

Patients and parents received detailed information on the characteristics of the assessment instruments and treatment options. All parents gave informed consent. The study was conducted in accordance with the Declaration of Helsinki. The methodology of the study was approved by the Ethics Committee of our Hospital (project identification code 153/2017).

### 2.2. Statistical Analyses

Descriptive analyses were used to describe demographic and clinical characteristics of the whole sample. Comparisons between groups were made using chi-square analyses on categorical variables and a *t*-test on continuous variables. Considering the large number of comparisons and the number of subjects in each group, our results are prone to both type I and type II errors, the false discovery rate (FDR) [51] correction of the *p*-values (implemented using the *p*.adjust function in R) [50] was applied for all these analyses. All these analyses were made using the Statistical Package for Social Science (SPSS) 25.0 for Windows.

We conducted a linear regression model with Children’s Global Assessment Scale (CGAS) scores (6th month) as a dependent variable in order to individuate putative predictors of outcomes of the pharmacological treatment. The predictors tested were CGAS score at baseline, age, gender, psychotherapy add-on, total number of internalizing disorders diagnosis and total number of externalizing disorders diagnosis. We did a post hoc power analysis to determine the statistical power of this model; we set these input parametres: sample size, 117; predictors, 6; effect size, 0.20; and α err probability, 0.05. The results indicated that the sample of this study had a power of 0.96. All models were analyzed using the Statistical Package for Social Science (SPSS) 25.0 for Windows. The false discovery rate (FDR) [51] correction of the *p*-values (implemented using the *p*.adjust function in R) [52]) was applied in this model.

## 3. Results

### 3.1. Clinical Characteristics and Gender Differences in the Whole Sample

The characteristics of the sample are summarized in Table 1. Comorbidity was almost the rule (89.7%), and most of the patients presented multiple comorbidities (mean comorbid diagnoses 3.0 ± 1.3). Anxiety disorders were reported in 62 patients (53.0%), and 29 patients (24.8%) had more than one anxiety disorder. Thirty-one patients (26.5%) had ADHD, and about 45% had ODD/CD. This was a sample of severely impaired patients, as evidenced not only by the baseline CGI-S and CGAS scores but also by the presence of severe suicidal ideation or suicidal attempts in 25 (21.4%) patients, whereas 32 patients (27.4%) presented a substance use disorder.

Regarding treatments, all patients received a pharmacological treatment, 81.2% with a mood stabilizer (with similar rates for lithium and valproic acid) and 61.5% with an SGA. Twenty-three patients, 19.7% of the total sample but 74.2% of the patients with comorbid ADHD, received MPH, while 25 (21.4%) received a SSRI. Of note, only 38.5% received monotherapy with mood stabilizers and 18.8% had SGA monotherapy, while a strong minority (42.7%) needed a combined mood stabilizer plus SGA pharmacotherapy. About half of the patients received additional psychotherapy.

It is noteworthy that a comparison between gender, after Benjamini–Hochberg correction, did not reveal significant differences in age at onset, severity, comorbidities (including ADHD, ODD, CD and substance use disorder) and rate of response to treatments, with pharmacotherapy and psychotherapy being similar in the two groups.

### 3.2. Comparison between Bipolar Disorders with Prepubertal and Postpubertal Onset

Thirty-nine patients (33.3%) had a BD onset before 12 years of age (“prepubertal onset”), while 78 (66.6%) had an onset after 12 years (“adolescent onset”) (Table 2). Patients with prepubertal-onset presented a higher rate of ADHD (and consequently a higher use of MPH) and of ODD/CD and a higher number of externalizing comorbidities, while the difference in SUD (15.4% in prepubertal onset vs. 34.6% in adolescent-onset) did not survive the Benjamini–Hochberg correction. Patients with adolescent-onset BD more frequently received mood stabilizers, while the use of SGAs did not differ between groups. Of note, at the 6-month follow-up, rates of responders, CGI-S, CGAS and CGI-I did not differ between groups.

### 3.3. Comparison between Bipolar Patients with or without ADHD

Thirty-one patients (26.6%) presented an associated ADHD (Table 3). Compared with the patients without ADHD, patients with ADHD presented an earlier age of onset of BD, and 76% had a prepubertal onset, compared to the 15% in BD patients without ADHD. They presented higher comorbidity with externalizing disorders, while baseline severity and response to treatments did not differ between groups.

### 3.4. Comparison between Bipolar Patients with or without Substance Use Disorders

Thirty-two patients (27.4%) fit the criteria for SUD (Table 4). Compared to patients without SUD, they were older and had a later onset of BD, while there was no significant difference between males and females. Externalizing comorbidities were more frequently associated with SUD. The severity and functional impairment at the 6-month follow-up as well as the rate of responders (21.2% vs. 63.1%) were negatively affected by SUD.

### 3.5. Comparison between Patients with or without Severe Suicidal Ideation or Behavior

Twenty-five patients (21.4%) presented severe suicidal ideation or behavior (Table 5). None of the selected measures differentiated patients with or without suicidality, including age at onset of BD; anxiety, ADHD or SUD comorbidities; and treatments.

Although baseline clinical severity, rate of multiple anxiety disorders (48% vs. 19.6%) and total number of comorbidities were higher in suicidal patients, differences did not survive the Benjamini–Hochberg correction.

### 3.6. Comparison between Bipolar Patients Responders and Nonresponders to Treatments

According to a double criterion of CGI-I scores 1 or 2 and an improvement of at least 30% of the CGAS, with a CGI-S score below 3 and C-GAS higher than 60 at the 6-month follow-up, 60 patients (51.3%) were considered responders (Table 6). The two groups received the same patterns of treatment (both pharmacotherapy and psychotherapy). Poorer response to treatments was associated with greater clinical severity (CGI-S) at baseline, and comorbidity was associated with CD and SUD. On the contrary, age at onset of BD and ADHD comorbidity did not affect the response to treatments.

Table 7 shows the results for the regression model testing putative predictors of response to treatment. The levels of the CGAS at baseline and the number of externalizing comorbidities predicted the levels of CGAS at follow-up: the more the number of externalizing comorbidities, the lower the levels of CGAS at the follow-up. This finding indicates that subjects with externalizing comorbidities are at risk for a worse outcome. Neither age, gender, psychotherapy add-on nor total number of internalizing comorbidities were significant predictors of change in CGAS scores. The regression model explained around 23% of the variance. Variance inflation (VIF) in this regression was 1.2.

## 4. Discussion

The aim of this naturalistic study, including a consecutive sample of children and adolescents referred with a manic or hypomanic episode, was to explore the effect of gender and age at onset (prepubertal and adolescent-onset) of BD, ADHD and SUD comorbidity on phenomenology, comorbidity and response to treatments. Furthermore, we explored possible elements associated with severe suicidal ideation or attempts and with poorer response to treatments. The sample consisted of patients referred for a pharmacological treatment and treated “as usual”, allowing us to explore possible elements associated with the effectiveness of treatments in daily clinical practice. Given the type of referral, the sample included severely impaired patients and about 90% of the patients presented psychiatric comorbidities. More specifically, two main patterns of comorbidity are confirmed, those with ADHD, with associated externalizing behaviors (about 50% of the patients), and those with anxiety disorders (about 50%, about half of them with at least two anxiety disorders) and obsessive compulsive disorder. These conditions may represent possible pathways to early-onset bipolarity [11].

In our sample, 81.2% of the patients received a mood stabilizer, only 18.8% received a first SGA monotherapy and only 38.5% remained on a mood stabilizer monotherapy, while a strong minority (42.7%) received a polypharmacy with both a mood stabilizer and an SGA. This is the consequence of our therapeutic approach, which considered as a first choice lithium or VPA, given their safer profile compared to SGAs, namely in terms of weight gain and metabolic side effects. The rate of polypharmacy, even if high, was lower compared to other studies exploring pharmacological management of referred bipolar youths. A study on the use of medications in 111 bipolar children and adolescents treated in the community in the USA showed that these patients were receiving 3.40 ± 1.48 medications, that 18% of these children were taking five or more medications currently, and that 77% had a trial with an antipsychotic [53]. In Pavuluri et al.’s [54] study, only 17.5% of bipolar children and adolescents without psychotic symptoms were effectively controlled by a monotherapy with a mood stabilizer for at least 6 months while 66.3% of those receiving a combination treatment with a mood stabilizer and an atypical antipsychotic were considered responders [54]. In the present study, SSRIs and MPH were used only after a stabilization with mood stabilizers and/or atypical antipsychotics, without destabilizing effects. Scheffer et al. [55] showed that, in youth with BD and comorbid ADHD firstly stabilized with valproate, the outcome was significantly improved by adding a stimulant. It is noteworthy that, in this sample of severely impaired patients, comorbidities did not differ between males and females, including disorders with typical prevalence in males, such as ADHD, CD and SUD. A comparison between prepubertal-onset and adolescent-onset BD showed that the similarities of the clinical expression are more striking than the discrepancies, consistent with previous studies [2,9], supporting the notion that the manifestations of BD remain stable over time. The role of age at onset of BD was evident mainly in relation to ADHD and, to a lesser degree, with ODD/CD comorbidities. This finding suggests that a marker of very early onset BD is the association with ADHD and externalizing disorders [3,9,10,12,24]. A higher risk of SUD in adolescence-onset BD has been previously reported [31] and should be further explored with larger samples and perspective studies. According to our data, an earlier onset as well as a lower chronological age do not influence the response to treatments, consistent with our previous findings [39,43].

These findings are consistent with those derived from the comparison between bipolar patients with or without ADHD. This association raised controversy, given the possibility of a chronic course and the overlap of symptoms, such as hyperactivity, impulsivity/aggressiveness, distractibility and emotional lability. In our study, comorbidity with ADHD is associated with an earlier onset and with a higher rate of externalizing disorders but it affects neither other comorbidities (including SUD) nor response to treatments. Differential diagnoses among early-onset mania, severe ADHD, ODD/CD with affective dysregulation and the cooccurrence of two or three of these disorders may be very difficult. The issue is not merely nosological, as a correct diagnosis has important implications for treatment options. The topic of comorbidity between ADHD, disruptive behavior disorders (particularly ODD) and bipolar spectrum disorders has been deeply explored, with the increasing impact of the concept of irritability and, more recently, of emotional dysregulation, that is an impaired regulation of emotional states, with excessive and inappropriate emotional expressions, high excitability and lability, temper outbursts, low tolerance to frustration and slow return to baseline [56]. This condition, firstly misconceived as a possible expression of BD comorbid to other clinical conditions, has been more recently and more adequately conceptualized as a neurodevelopmental condition of early severe dysregulation of emotions and behavior, not completely fitting any of the current clinical categories (i.e., ADHD, ODD and mood disorders), although they share features of all these domains. In a cross-sectional community study, mood lability, a concept closely related to emotional dysregulation, was strongly associated with comorbidity between internalizing and externalizing disorders, suggesting that it could be a shared risk factor for both disorders [57]. In a developmental perspective, children with ODD and emotional dysregulation at age 8 were found to be at higher risk of presenting mood disorders at age 14 [58].

Comorbidity with SUD is associate with greater severity and impairment, as expected, although it cannot be defined in the direction of this association (more severe patients are at higher risk of SUD, and/or SUD increases the severity of the affective and behavioral symptoms). The comorbidity of externalizing disorders (ADHD and ODD/CD) in BD youth with SUD has been previously reported [15,59] and probably represents the higher risk factor. These findings, including the higher rates of SUD in adolescent-onset BD, are comparable with those reported in Wilens et al.‘s study [31], that found that both CD and BD are independent risk factors for adolescent-onset substance abuse. However, Wilens et al. [31] did not find a greater additive risk of substance abuse in youths with the combination of BD and CD. The lower response to treatments in patients with SUD may be related to the effects of substances on mood, to the greater severity of the clinical picture, or to the lower compliance to treatments.

The research of specific elements associated with suicidality in BD patients was disappointing, as none of the selected measures resulted in differentiating patients with or without suicidality, including age at onset of BD; anxiety, ADHD or SUD comorbidities; and pharmacological or psychological treatments. It may be possible that a larger sample may support the possible role of specific elements, including baseline clinical severity, rate of multiple anxiety disorders (48% vs. 19.6%) and the total number of comorbidities, which were no more significant after correction. More specifically, multiple anxiety disorders as a risk factor for suicidality in BD patients deserve a closer inspection [30].

Finally, both the comparison between responders and nonresponders and the logistic regressions underline the role of baseline severity and externalizing disorders as critical elements indicating the response to treatment. In previous studies [39,42,45], comorbidity with CD was the most important negative prredicitor of outcome and comorbid ADHD (odd ratio 2.30) and baseline CGI-S score were also significant.

Different mechanisms can be involved in treatment resistance of bipolar subjects with comorbid externalizing disorders. BD plus externalizing disorders may represent a specific subtype with earlier-onset and resistance to traditional anti-manic and mood stabilizing drugs [59]. Poorer treatment response in BD with cooccurring externalizing disorders may be also accounted for by more problematic compliance to treatments. According to Carlson et al. [60], 61.5% of bipolar youths with comorbid externalizing disorder—compared to 22.2% in patients without externalizing disorder—discontinued the medication during follow-up and more than half of them had at least one recurrence. Their early course was negative; their global functioning at 24-month follow-up was low; and 50% of them, compared to 0% in the non-externalizing patients, were unable to interrupt the abuse of illicit drugs or alcohol during this period.

Our study presents several limitations. The main limitation is the lack of a control group and the use of mixed pharmacological treatments. Only patients referred to our third-level university hospital for severe symptomatology and pharmacological treatment were included, and they may represent a subgroup of more severely impaired subjects in terms of clinical presentation, pattern of comorbidity and response to treatments. The trained child psychiatrist was the same one who participated in the subsequent assessment procedures on the same subjects, and thus, he was not blind to the nature of the diagnosis. The age of onset of BD was assessed retrospectively, based on historical information and previous clinical reports. Only a selected number of features were considered relevant, and the diagnostic exploration did not include other potentially important elements. Environmental and personality trait variables may have been of interest [61], but they were not included in our study. Furthermore, the period of follow-up was limited to 6 months; a more extended observation would have been useful to better ascertain clinical characteristics and response to treatments. We have used as outcome measures CGI-S, C-GAS and CGI-I, not a specific measure of BD symptoms’ severity and improvement. However, CGI-I is the criterion according to which usually clinicians decide treatment strategies, i.e., to continue or change a pharmacotherapy. In this situation, the clinical picture as a whole is more reliably captured by a global measure, considered to best fit the practical goals of our study.

Our findings describe an unselected sample of consecutive children and adolescents with BD treated in an “ordinary” clinical setting, which may actually be one of the strengths of our study. No exclusion criteria were applied (except for intellectual disability), and all comorbid conditions, which are often excluded in controlled trials but represent the rule in clinical settings, were included in the study. Furthermore, all the patients were treated as needed (mono- or polypharmacy) and followed-up in a routinary clinical setting. We submit that long-term naturalistic perspective studies might represent an important source of information regarding the effectiveness of treatment over extended periods of time under ordinary clinical conditions.

## Figures and Tables

**Table 1:** Clinical characteristics of the sample.

	Total (*N* = 117)	Gender		t or χ^2^ (df)
		Males (*N* = 69)	Females (*N* = 48)	
Age, mean (SD)	14.59 (2.51)	14.51 (2.76)	14.65 (2.13)	0.29 (115)
Age at onset, mean (SD)	12.55 (2.99)	12.41 (3.09)	12.82 (2.72)	0.74 (115)
Prepubertal-onset, *N* (%)	39 (33.30)	27 (39.10)	11 (22.90)	2.69 (1)
CGI Severity (baseline) mean (SD)	5.56 (.74)	5.59 (.73)	5.44 (.72)	1.10 (115)
CGI Severity (6th month) mean (SD)	3.27 (1.29)	3.34 (1.37)	3.17 (1.17)	0.79 (115)
CGAS Score (baseline), mean (SD)	37.89 (4.74)	38.32 (4.63)	37.53 (4.88)	0.89 (115)
CGAS Score (6th month), mean (SD)	52.72 (9.30)	52.36 (9.40)	54.11 (9.79)	0.97 (115)
CGI Improvement, mean (SD)	2.44 (.81)	2.46 (.79)	2.36 (.82)	0.66 (115)
Responders, *N* (%)	60 (51.30)	34 (49.30)	26 (54.17)	0.11 (1)
Suicidality *N* (%)	25 (21.40)	16 (23.20)	9 (18.70)	0.120 (1)
Lifetime comorbidity, *N* (%)				
GAD	32 (27.40)	21 (30.40)	11 (22.90)	0.47 (1)
Social phobia	17 (14.60)	12 (17.40)	5 (10.40)	1.28 (1)
Separation Anxiety	22 (18.80)	13 (18.80)	9 (18.70)	0.05 (1)
Panic Disorder-Agoraphobia	15 (12.80)	5 (7.50)	9 (18.70)	2.55 (1)
Simple Phobias	14 (12.00)	10 (14.50)	3 (6.20)	1.20 (1)
Anxiety Disorders	63 (53.80)	39 (56.50)	23 (47.90)	0.53 (1)
Multiple Anxiety Disorders	30 (25.60)	19 (27.50)	11 (22.90)	0.12 (1)
*N* * of Anxiety Disorders	0.95 (1.160)	0.96 (1.10)	0.96 (1.28)	0.00 (115)
OCD	21 (17.90)	17 (24.60)	4 (8.20)	4.06 (1)
Tic	4 (3.40)	4 (5.80)	0 (0.00)	1.39 (1)
ADHD	31 (26.50)	20 (29.00)	10 (20.40)	0.60 (1)
ODD	34 (29.10)	19 (27.50)	14 (28.60)	0.00 (1)
CD	25 (21.40)	11 (15.90)	13 (26.50)	1.53 (1)
ODD + CD	51 (43.60)	28 40.60)	21 (42.80)	0.02 (1)
Borderline Personality Disorder	16 (13.70)	4 (5.80)	10 (20.40)	4.73 (1)
Substance Use Disorder	33 (28.20)	14 (20.30)	8 (16.30)	0.09 (1)
Total Comorbidities	2.30 (1.44)	2.23 (1.48)	2.36 (1.43)	0.47 (115)
Total Internalizing Disorders	1.08 (1.23)	1.12 (1.32)	1.04 (1.40)	0.31 (115)
Total Externalizing Disorders	0.72 (0.71)	0.70 (0.71)	0.73 (0.69)	0.23 (115)
Pharmacological Treatment, *N* (%)				
SSRI	25 (21.40)	14 (20.30)	11 (22.90)	0.00 (1)
Mood stabilizers	95 (81.20)	58 (84.10)	34 (69.40)	2.21 (1)
Valproic Acid	57 (48.70)	35 (50.70)	20 (40.80)	0.60 (1)
Lithium	54 (46.20)	36 (59.20)	16 (32.60)	3.34 (1)
SGAs	72 (61.50)	41 (59.40)	29 (59.20)	0.01 (1)
Stimulants	23 (19.70)	16 (23.20)	7 (14.30)	0.84 (1)
MS only	45 (38.50)	28 (40.60)	16 (32.60)	0.36 (1)
Antipsychotic only	22 (18.80)	11 (15.90)	11 (22.90)	0.50 (1)
MS + Antipsychotics	50 (42.70)	30 (43.50)	18 (36.70)	0.21 (1)
Psychotherapy, *N* (%)	58 (49.60)	34 (49.30)	23 (46.90)	0.00 (1)

*Note*. CGI: Clinical Global Impression scale; CGAS: Children’s Global Assessment Scale; GAD: Generalized Anxiety Disorder; OCD: Obsessive Compulsive Disorder; ODD: Oppositional-Defiant Disorder; CD: Conduct Disorder; SGAs: Second Generation Antipsychotics; MS: Mood Stabilizers. False discovery rate (FDR; [51]) correction of the *p*-values (implemented using the *p*.adjust function in R) was applied. * p ≤ 0.05.

**Table 2:** Comparisons between prepubertal (*N* = 39) and adolescent-onset (*N* = 78).

	Prepub.-Onset (*N* = 39)	Adolesc.-Onset (*N* = 78)	t or χ^2^ (df)
Gender, Males, *N* (%)	27 (69.20)	42 (53.80)	1.95 (1)
Age, mean (SD)	12.39 (2.65)	15.70 (1.53)	8.56 (115) ***
Age at onset, mean (SD)	8.98 (1.55)	14.33 (1.62)	17.08 (115) ***
CGI Severity (baseline) mean (SD)	5.61 (.63)	5.53 (.78)	0.56 (115)
CGI Severity (6th month) mean (SD)	3.43 (1.50)	3.19 (1.17)	0.95 (115)
CGAS Score (baseline), mean (SD)	37.69 (4.46)	37.99 (4.99)	0.32 (115)
CGAS Score (6th month), mean (SD)	52.23 (1.11)	52.96 (8.92)	0.51 (115)
CGI Improvement, mean (SD)	2.49 (0.85)	2.42 (.79)	0.44 (115)
Responders, *N* (%)	21 (53.80)	39 (50.00)	0.04 (1)
Suicidality, *N* (%)	10 (25.60)	15 (19.20)	0.31 (1)
Lifetime comorbidity, *N* (%)			
GAD	10 (25.60)	22 (28.20)	0.05 (1)
Social phobia	9 (23.10)	13 (16.70)	0.34 (1)
Separation Anxiety	9 (23.10)	13 (16.70)	0.34 (1)
Panic Disorder-Agoraphobia	4 (10.20)	11 (14.10)	0.09 (1)
Simple Phobias	8 (20.50)	6 (7.70)	2.93 (1)
Anxiety Disorders	23 (59.99)	40 (51.30)	0.35 (1)
Multiple Anxiety Disorders	23 (59.00)	40 (51.30)	0.35 (1)
*N* * of Anxiety Disorders	1.08 (1.26)	0.88 (1.10)	0.88 (115)
OCD	9 (23.10)	12 (15.40)	0.059 (1)
Tic	2 (5.10)	2 (2.60)	0.32 (1)
ADHD	25(12.80)	6 (7.70)	36.93 (1) ***
ODD	17 (43.60)	17 (21.80)	4.98 (1)
CD	5 (12.80)	13 (16.70)	0.07 (1)
ODD + CD	21 (53.80)	20 (25.60)	7.89 (1) *
Borderline Personality Disorder	3 (7.70)	13 (16.70)	0.10 (1)
Substance Use Disorder	6 (15.40)	27 (34.60)	3.85 (1)
Total Comorbidities	2.67 (1.49)	2.11 (1.39)	2.00 (115)
Total Internalizing Disorders	1.23 (1.31)	1.00 (1.18)	0.96 (115)
Total Externalizing Disorders	1.13 (.80)	0.51 (.55)	4.91 (115) ***
Pharmacological Treatment, *N* (%)			
SSRI	9 (23.10)	16 (20.50)	0.01 (1)
Mood stabilizers	20 (51.30)	66 (84.60)	13.17 (1) ***
Valproic Acid	10 (25.60)	37 (47.40)	4.27 (1)
Lithium	14 (10.20)	40 (51.30)	5.98 (1)
SGAs	26 (66.60)	46 (59.00)	0.37 (1)
Stimulants	17 (43.60)	6 (7.70)	19.00 (1) ***
MS only	13 (33.30)	32 (41.00)	0.31 (1)
Antipsychotic only	10 (25.60)	12 (15.40)	1.18 (1)
MS + Antipsychotics	16 (41.00)	34 (43.60)	0.00 (1)
Psychotherapy, *N* (%)	24 (61.50)	34 (43.60)	2.87 (1)

*Note*. CGI: Clinical Global Impression scale; CGAS: Children’s Global Assessment Scale; GAD: Generalized Anxiety Disorder; OCD: Obsessive Compulsive Disorder; ODD: Oppositional-Defiant Disorder; CD: Conduct Disorder; SGAs: Second Generation Antipsychotics; MS: Mood Stabilizers. False discovery rate (FDR; [51] correction of the *p*-values (implemented using the *p*.adjust function in R) was applied. * p ≤ 0.05; *** *p* ≤ 0.001.

**Table 3:** Comparison between patients with (*N* = 31) and without (*N* = 86) attention deficit hyperactivity disorder (ADHD).

	BD + ADHD (*N* = 31)	BD w/out ADHD (*N* = 86)	t or χ^2^ (df)
Gender, Maled, *N* (%)	20 (64.50)	49 (56.90)	0.27 (1)
Age, mean (SD)	12.60 (2.84)	15.33 (1.92)	5.93 (115) ***
Age at onset, mean (SD)	9.91 (2.57)	13.50 (2.54)	6.73 (115) ***
Prepubertal onset, *N* (%)	25 (78.10)	14 (16.30)	39.63 (1) ***
CGI Severity (baseline) mean (SD)	5.52 (0.62)	5.53 (.78)	0.06 (115)
CGI Severity (6th month) mean (SD)	3.48 (1.41)	3.19 (1.17)	1.12 (115)
CGAS Score (baseline), mean (SD)	38.54 (4.95)	37.99 (4.99)	0.53 (115)
CGAS Score (6th month), mean (SD)	50.87 (10.02)	52.96 (8.92)	1.08 (115)
CGI Improvement, mean (SD)	2.61 (0.83)	2.42 (0.79)	1.13 (115)
Responders, *N* (%)	13 (41.90)	47 (54.60)	1.01 (1)
Suicidality, *N* (%)	8 (25.80)	17 (19.80)	0.20 (1)
Lifetime comorbidity, *N* (%)			
GAD	7 (22.60)	25 (29.10)	0.21 (1)
Social phobia	3 (9.70)	14 (16.30)	0.36 (1)
Separation Anxiety	5 (16.10)	17 (19.80)	0.03 (1)
Panic Disorder-Agoraphobia	4 (12.90)	11 (12.80)	0.09 (1)
Simple Phobias	6 (19.30)	8 (9.30)	1.34 (1)
Anxiety Disorders	17 (54.80)	46 (55.40)	0.01 (1)
Multiple Anxiety Disorders	6 (19.40)	24 (28.90)	0.43 (81)
*N* * of Anxiety Disorders	1.00 (1.27)	0.95 (1.14)	0.20 (115)
OCD	3 (9.70)	18 (21.70)	1.27 (1)
Tic	2 (6.40)	2 (2.30)	0.26 (1)
ODD	15 (48.40)	19 (22.1)	6.42 (1)
CD	4 (12.90)	21 (24.40)	1.18 (1)
ODD + CD	18 (58.10)	33 (38.40)	2.84 (10)
Borderline Personality Disorder	2 (6.40)	14 (16.30)	1.12 (1)
Substance Use Disorder	8 (25.80)	28 (32.60)	0.22 (1)
Total Comorbidities	2.77 (1.31)	2.13 (1.45)	2.16 (115)
Total Internalizing Disorders	1.03 (1.33)	1.09 (1.19)	0.23 (115)
Total Externalizing Disorders	1.48 (0.57)	0.44 (0.52)	9.31 (115) ***
Pharmacological Treatment, *N* (%)			
SSRI	5 (16.10)	20 (23.30)	0.33 (1)
Mood stabilizers	24 (77.40)	71 (82.60)	0.13 (1)
Valproic Acid	16 (51.60)	41 (47.70)	0.03 (1)
Lithium	12 (38.70)	42 (48.80)	0.58 (1)
SGAs	20 (64.50)	52 (60.40)	0.03 (1)
Stimulants	23 (74.20)	0 (0.00)	74.79 (1) ***
MS only	11 (35.50)	34 (39.50)	0.44 (1)
Antipsychotic only	7 (22.60)	15 (17.40)	0.13 (1)
MS + Antipsychotics	13 (41.90)	37 (43.00)	0.01 (1)
Psychotherapy, *N* (%)	17 (54.80)	41 (47.70)	0.22 (1)

*Note*. CGI: Clinical Global Impression scale; CGAS: Children’s Global Assessment Scale; GAD: Generalized Anxiety Disorder; OCD: Obsessive Compulsive Disorder; ODD: Oppositional-Defiant Disorder; CD: Conduct Disorder; SGAs: Second Generation Antipsychotics; MS: Mood Stabilizers. False discovery rate (FDR; [51] correction of the *p*-values (implemented using the *p*.adjust function in R) was applied. * p ≤ 0.05; *** *p* ≤ 0.001.

**Table 4:** Comparisons between patients with (*N* = 33) and without substance use disorder (*N* = 84).

	BD + SUD (*N* = 33)	BD w/o SUD (*N* = 84)	t or χ^2^ (df)
Gender, Males, *N* (%)	14 (42.40)	55 (65.50)	4.29 (1)
Age, mean (SD)	15.60 (1.93)	14.20 (2.61)	2.79 (115) *
Age at onset, mean (SD)	13.76 (2.24)	12.07 (3.12)	2.83 (115) *
Prepubertal onset, *N* (%)	6 (18.20)	33 (39.30)	3.85 (1)
CGI Severity (baseline), mean (SD)	5.76 (0.79)	5.48 (0.70)	1.88 (115)
CGI Severity (6th month), mean (SD)	3.73 (0.91)	3.09 (1.38)	2.46 (115) ***
CGAS Score (baseline), mean (SD)	36.88 (4.13)	38.29 (4.92)	1.46 (115)
CGAS Score (6th month), mean (SD)	47.18 (7.89)	54.89 (8.94)	4.33 (115)
CGI Improvement, mean (SD)	2.85 (0.66)	2.28 (0.81)	3.60 (115) ***
Responders, *N* (%)	7 (21.20)	53 (63.10)	15.00 (1) ***
Suicidality, *N* (%)	10 (30.30)	15 (17.80)	1.51 (1)
Lifetime comorbidity, *N* (%)			
GAD	11 (33.30)	21 (25.00)	0.46 (1)
Social phobia	7 (21.20)	10 (11.90)	0.99 (1)
Separation Anxiety	9 (27.30)	13 (15.50)	1.46 (1)
Panic Disorder-Agoraphobia	8 (24.20)	7 (8.30)	4.04 (1)
Simple Phobias	4 (12.10)	10 (11.90)	0.08 (1)
Anxiety Disorders	21 (63.60)	42 (50.00)	1.69 (1)
Multiple Anxiety Disorders	11 (33.30)	19 (22.60)	0.92 (1)
*N* * of Anxiety Disorders	1.00 (1.27)	0.85 (1.09)	0.64 (115)
OCD	6 (18.20)	15 (17.80)	0.05 (1)
Tic	1 (3.00)	3 (3.60)	0.18 (1)
ADHD	5 (15.10)	26 (31.00)	1.83 (1)
ODD	3 (9.10)	19 (22.60)	2.02 (1)
CD	18 (54.50)	21 (25.00)	8.02 (1) *
ODD + CD	20 (60.60)	33 (39.30)	3.53 (1)
Borderline Personality Disorder	8 (24.20)	8 (9.50)	3.19 (1)
Total Comorbidities	2.77 (1.31)	1.93 (1.35)	0.28 (115)
Total Internalizing Disorders	1.03 (1.33)	0.99 (1.17)	0.16 (115)
Total Externalizing Disorders	1.48 (.57)	0.74 (0.75)	5.11 (115) ***
Pharmacological Treatment, *N* (%)			
SSRI	8 (24.20)	17 (20.20)	0.05 (1)
Mood stabilizers	24 (72.70)	71 (84.50)	1.46 (1)
Valproic Acid	15 (45.40)	42 (50.00)	0.06 (1)
Lithium	17 (51.50)	37 (44.00)	0.27 (1)
SGAs	25 (45.40)	47 (55.90)	3.13 (1)
Stimulants	4 (12.10)	19 (22.60)	1.05 (1)
MS only	8 (24.20)	37 (44.00)	3.13 (1)
Antipsychotic only	9 (27.30)	13 (15.50)	1.46 (1)
MS + Antipsychotics	16 (48.50)	34 (40.50)	0.34 (1)
Psychotherapy, *N* (%)	16 (48.50)	42 (50.00)	0.00 (1)

*Note*. CGI: Clinical Global Impression scale; CGAS: Children’s Global Assessment Scale; GAD: Generalized Anxiety Disorder; OCD: Obsessive Compulsive Disorder; ODD: Oppositional-Defiant Disorder; CD: Conduct Disorder; SGAs: Second Generation Antipsychotics; MS: Mood Stabilizers. False discovery rate (FDR; [51] correction of the *p*-values (implemented using the *p*.adjust function in R) was applied. * p ≤ 0.05; *** *p* ≤ 0.001.

**Table 5:** Comparisons between patients with (*N* = 25) and without suicidality (*N* = 92).

	BD + Suicidality (*N* = 25)	BD w/out Suicidality (*N* = 92)	t or χ^2^ (df)
Gender, Maled, *N* (%)	16 (64.00)	53 (58.20)	0.12 (1)
Age, mean (SD)	15.24 (2.44)	14.42 (2.51)	1.46 (115)
Age at onset, mean (SD)	12.84 (3.27)	12.47 (2.92)	0.55 (115)
Prepubertal onset, *N* (%)	10 (40.00)	29 (31.90)	0.31 (1)
CGI Severity (baseline) mean (SD)	5.84 (0.75)	5.48 (0.72)	2.20 (115)
CGI Severity (6th month) mean (SD)	3.44 (1.42)	3.23 (1.26)	0.72 (115)
CGAS Score (baseline), mean (SD)	37.76 (3.62)	37.92 (5.02)	0.15 (115)
CGAS Score (6th month), mean (SD)	52.00 (9.77)	54.89 (8.94)	−1.40 (115)
CGI Improvement, mean (SD)	2.44 (0.85)	2.45 (0.80)	0.05 (115)
Responders, *N* (%)	12 (40.00)	48 (52.70)	0.02 (1)
Lifetime comorbidity, *N* (%)			
GAD	10 (40.00)	22 (23.90)	1.81 (1)
Social phobia	5 (20.00)	12 (13.00)	0.31 (1)
Separation Anxiety	9 (36.00)	13 (14.10)	4.81 (1)
Panic Disorder-Agoraphobia	6 (24.00)	9 (9.80)	2.40 (1)
Simple Phobias	3 (12.00)	11 (12.00)	0.12 (1)
Anxiety Disorders	18 (72.00)	45 (48.90)	3.34 (1)
Multiple Anxiety Disorders	12 (48.00)	18 (19.60)	6.91 (1)
*N* * of Anxiety Disorders	1.44 (1.19)	0.82 (1.12)	2.42 (115)
OCD	3 (12.00)	18 (19.60)	0.34 (1)
Tic	0 (0.00)	4 (4.30)	0.19 (1)
ADHD	8 (32.00)	23 (25.00)	0.47 (1)
ODD	8 (32.00)	26 (28.3)	0.01 (1)
CD	6 (24.00)	19 (20.60)	0.01 (1)
ODD + CD	11 (44.00)	40 (43.50)	0.03 (1)
Borderline Personality Disorder	5 (20.00)	11 (12.00)	0.50 (1)
Substance Abuse Disorder	10 (40.00)	23 (25.00)	1.51 (1)
Total Comorbidities	2.92 (1.41)	2.13 (1.41)	2.48 (115)
Total Internalizing Disorders	1.52 (1.19)	0.96 (1.21)	2.06 (115)
Total Externalizing Disorders	0.76 (0.72)	0.71 (0.70)	0.31 (115)
Pharmacological Treatment, *N* (%)			
SSRI	5 (20.00)	20 (21.70)	0.01 (1)
Mood stabilizers	19 (76.00)	76 (82.60)	0.21 (1)
Valproic Acid	8 (32.00)	41 (44.60)	0.81 (1)
Lithium	14 (56.00)	40 (43.50)	0.79 (1)
SGAs	14 (56.00)	58 (63.00)	2.21 (1)
Stimulants	7 (28.00)	16 (17.40)	0.81 (1)
MS only	11 (44.00)	34 (36.90)	0.17 (1)
Antipsychotic only	6 (24.00)	16 (17.40)	0.21 (1)
MS + Antipsychotics	8 (32.00)	42 (45.60)	0.99 (1)
Psychotherapy, *N* (%)	15 (60.00)	43 (46.70)	0.90 (1)

*Note*. CGI: Clinical Global Impression scale; CGAS: Children’s Global Assessment Scale; GAD: Generalized Anxiety Disorder; OCD: Obsessive Compulsive Disorder; ODD: Oppositional-Defiant Disorder; CD: Conduct Disorder; SGAs: Second Generation Antipsychotics; MS: Mood Stabilizers. False discovery rate (FDR; [51] correction of the *p*-values (implemented using the *p*.adjust function in R) was applied. * p ≤ 0.05.

**Table 6:** Comparison between patients responders (*N* = 60) and nonresponders (*N* = 57).

	Responders (*N* = 60)	Nonresponders (*N* = 57)	t or χ^2^ (df)
Gender, Maled, *N* (%)	34 (56.70)	35 (61.40)	0.11 (1)
Age, mean (SD)	14.08 (2.72)	15.13 (2.16)	2.30 (115)
Age at onset, mean (SD)	12.43 (3.01)	12.67 (2.99)	0.43 (115)
Prepubertal onset, *N* (%)	21 (35.00)	18 (31.60)	0.04 (1)
CGI Severity (baseline) mean (SD)	5.37 (0.69)	5.75 (0.74)	2.87 (115) *
CGI Severity (6th month) mean (SD)	2.37 (0.64)	4.00 (1.00)	10.81 (115) ***
CGAS Score (baseline), mean (SD)	38.43 (4.73)	37.32 (4.72)	1.27 (115)
CGAS Score (6th month), mean (SD)	60.42 (1.67)	44.61 (6.78)	17.52 (115) ***
CGI Improvement, mean (SD)	1.83 (0.46)	3.09 (0.58)	13.82 (115) ***
Suicidality, *N* (%)	14 (23.30)	13 (22.80)	0.02 (1)
Lifetime comorbidity, *N* (%)			
GAD	18 (30.00)	14 (24.60)	0.20 (1)
Social phobia	14 (23.30)	14 (24.60)	0.00 (1)
Separation Anxiety	11 (18.30)	11 (19.30)	0.01 (1)
Panic Disorder-Agoraphobia	9 (15.00)	6 (10.50)	0.20 (1)
Simple Phobias	8 (13.30)	6 (10.50)	0.03 (1)
Anxiety Disorders	33 (55.00)	29 (50.90)	0.07 (1)
Multiple Anxiety Disorders	16 (26.70)	14 (24.60)	0.00 (1)
*N* * of Anxiety Disorders	1.00 (1.15)	0.89 (1.17)	0.51 (115)
OCD	12 (20.00)	9 (15.80)	0.12 (1)
Tic	1 (1.70)	3 (5.30)	0.31 (1)
ADHD	13 (21.70)	18 (31.60)	1.01 (1)
ODD	19 (31.70)	15 (26.30)	0.19 (1)
CD	5 (8.30)	20 (35.10)	10.91 (1) ***
ODD + CD	23 (38.30)	28 (49.10)	2.81 (1)
Borderline Personality Disorder	7 (11.70)	9 (15.80)	0.14 (1)
Substance Abuse Disorder	7 (11.70)	26 (45.69)	0.15 (1) ***
Total Comorbidities	2.08 (1.43)	2.53 (1.43)	1.70 (115)
Total Internalizing Disorders	1.15 (1.21)	1.00 (1.23)	0.66 (115)
Total Externalizing Disorders	0.58 (0.62)	0.86 (0.77)	−2.01 (115)
Pharmacological Treatment, *N* (%)			
SSRI	16 (26.70)	9 (15.80)	1.46 (1)
Mood stabilizers	47 (78.30)	48 (84.20)	0.33 (1)
Valproic Acid	27 (45.00)	30 (52.60)	0.41 (1)
Lithium	27 (45.00)	27 (47.40)	0.01 (1)
SGAs	33 (55.00)	39 (68.40)	1.69 (1)
Stimulants	8 (13.30)	15 (26.30)	2.35 (1)
MS only	27 (45.00)	18 (31.60)	1.69 (1)
Antipsychotic only	13 (21.70)	9 (15.80)	0.33 (1)
MS + Antipsychotics	20 (33.30)	30 (52.60)	3.69 (1)
Psychotherapy, *N* (%)	31 (51.70)	27 (47.40)	0.08 (1)

*Note*. CGI: Clinical Global Impression scale; CGAS: Children’s Global Assessment Scale; GAD: Generalized Anxiety Disorder; OCD: Obsessive Compulsive Disorder; ODD: Oppositional-Defiant Disorder; CD: Conduct Disorder; SGAs: Second Generation Antipsychotics; MS: Mood Stabilizers. False discovery rate (FDR; [51]) correction of the *p*-values (implemented using the *p*.adjust function in R) was applied. * p ≤ 0.05; *** *p* ≤ 0.001.

**Table 7:** Linear regression model with CGAS scores (6th month) as the dependent variable.

	B	Std. Error	β	*p*
CGAS Score (baseline)	0.694	0.165	0.354	0.000
Age	−0.083	0.027	−0.269	0.004
Gender	−1.997	1.543	−0.106	0.252
Psychotherapy	1.946	1.542	0.105	0.252
Total Internalizing Disorders	−0.692	0.642	−0.091	0.284
Total Externalizing Disorders	−4.990	1.183	−0.378	0.000

*Note*. CGAS: Children’s Global Assessment Scale. B: unstandardized beta. False discovery rate (FDR; [51]) correction of the *p*-values (implemented using the *p*.adjust function in R) was applied for all predictors. R = 0.523; R^2^ = 0.273; and adjusted R^2^ = 0.233.
